# SIRTain regulators of premature senescence and accelerated aging

**DOI:** 10.1007/s13238-015-0149-1

**Published:** 2015-04-25

**Authors:** Shrestha Ghosh, Zhongjun Zhou

**Affiliations:** 1 Department of Biochemistry, The University of Hong Kong, Hong Kong, China; 2 Shenzhen Institute of Innovation and Technology, The University of Hong Kong, Hong Kong, China

**Keywords:** sirtuins, senescence, premature aging, longevity

## Abstract

The sirtuin proteins constitute class III histone deacetylases (HDACs). These evolutionarily conserved NAD^+^-dependent enzymes form an important component in a variety of cellular and biological processes with highly divergent as well as convergent roles in maintaining metabolic homeostasis, safeguarding genomic integrity, regulating cancer metabolism and also inflammatory responses. Amongst the seven known mammalian sirtuin proteins, SIRT1 has gained much attention due to its widely acknowledged roles in promoting longevity and ameliorating age-associated pathologies. The contributions of other sirtuins in the field of aging are also gradually emerging. Here, we summarize some of the recent discoveries in sirtuins biology which clearly implicate the functions of sirtuin proteins in the regulation of premature cellular senescence and accelerated aging. The roles of sirtuins in various cellular processes have been extrapolated to draw inter-linkage with anti-aging mechanisms. Also, the latest findings on sirtuins which might have potential effects in the process of aging have been reviewed.

## Introduction

**Figure 1:**
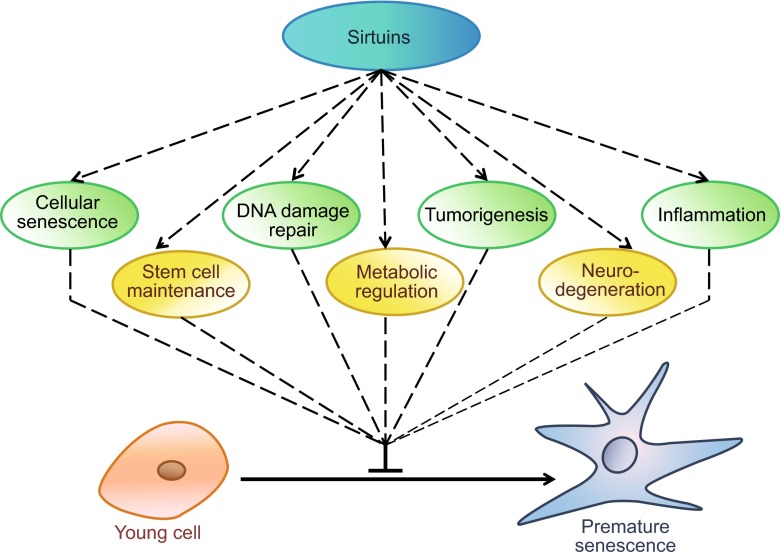
Schematic representation showing the regulation of premature aging by sirtuins via multiple biological processes

The phenomenon of premature aging has intrigued researchers all over the globe. Given that the primary motive of nearly all biomedical research is to promote healthspan of individuals or target diseases to improve health of patients, study of the aging process and other pathologies associated with it has ignited huge interest in dissecting the molecular pathways contributing to this process. In this scenario, premature aging disorders in humans and the various animal model systems recapitulating the phenotypes of accelerated aging and cellular senescence constitute a major area of interest in understanding the intricacies of the process of aging. The field of premature aging has garnered immense interest also because of the emerging number of premature aging disorders observed in humans, with a significant proportion of the syndromes originating from mutations in particular genes. For example, laminopathy-based premature aging syndromes (collectively known as laminopathies) originate from mutations in the *LMNA* gene or *ZMPSTE24* gene (Schreiber and Kennedy, 2013). Also, mutation or deletion of genes involved in DNA damage repair or chromatin remodeling, result in premature aging phenotypes (Ghosh and Zhou, 2014). In this regard, the silent information regulator proteins (SIRT) or the sirtuins have displayed intricate roles in promoting anti-aging effects via modulation of a spectrum of biological processes ranging from genomic maintenance, metabolic regulation, tumor suppression, inflammation and others (Fig. 1) (Choi and Mostoslavsky 2014). The sirtuins are evolutionarily conserved NAD^+^-dependent deacylases and ADP-ribosyltransferases (Saunders and Verdin 2007). In mammals, seven sirtuin proteins (SIRT1–7) have been identified as of now with conserved central catalytic core domains flanked by differing amino and carboxyl termini, which confer individuality to the seven sirtuins in terms of structure, cellular localization and functioning (Saunders and Verdin 2007). In this review, we have analyzed the varying roles of sirtuins with implications in the process of premature cellular senescence and accelerated aging.

## SIRT1

SIRT1 is by far the most extensively studied mammalian sirtuin. Its multifaceted roles in a spectrum of pivotal biological functions, such as metabolic regulation, tumor suppression, apoptosis and other stress response signaling pathways, and also aging processes, have brought this sirtuin to limelight (Giblin et al., 2014). SIRT1 has garnered further attention by being involved in longevity expansion and also premature aging via regulation of multiple signaling pathways and cellular processes (Rehan et al., 2014). Some of the recent advancements which solidify the importance of SIRT1 in premature senescence and accelerated aging are discussed below:

## Cellular senescence

Mammalian SIRT1, the closest homologue of yeast sirt2 protein, has time and again been linked to premature cellular senescence, one of the critical processes contributing to accelerated aging. The involvement of SIRT1 in modulating cellular senescence was identified with its deacetylation of p53 and eventual attenuation of promyelocytic leukemia protein (PML)-mediated premature cellular senescence (Langley et al., 2002). Loss of SIRT1 level or activity in human umbilical vein endothelial cells (HUVECs) results in premature senescence-associated phenotypes, such as enhanced β-galactosidase (SA-β-gal) activity, persistent growth arrest and also flattened cellular morphology (Ota et al., 2007). This suggests a protective role of SIRT1 in preventing endothelial dysfunction, which is one of the resulting features of cellular senescence. The role of SIRT1 as a guardian against endothelial cellular senescence is further bolstered by the finding that inhibition of SIRT1 expression by miR-217 promotes cellular senescence in the endothelial cells (Menghini et al., 2009). Upregulation of SIRT1 expression by peroxisome proliferator-activated receptor (PPAR)δ also downregulates early senescence in angiotensin (Ang) II-treated human coronary artery endothelial cells (HCAECs) (Kim et al., 2012). It is reported that hyperglycemia promotes premature senescence in vascular endothelial cells by downregulating SIRT1 expression levels (Mortuza et al., 2013). Further, the protective functions of cilostazol (a selective PDE3 inhibitor) in oxidative stress-induced premature senescence in endothelial cells, has been traced back to the upregulation of SIRT1 expression (Ota et al., 2008). On the other hand, the role of persistent insulin-like growth factor (IGF) treatment in triggering premature cellular senescence in a p53-dependent manner is well known (Salvioli et al., 2009). A recent study unraveled that this phenomenon results in attenuation of SIRT1 deacetylase functioning, causing enhanced p53 acetylation and stabilization, thus bringing about premature cellular senescence (Tran et al., 2014). Taken together, SIRT1 stands as a major protector against the progression of cardiovascular diseases which are triggered by endothelial dysfunctioning.

Apart from protecting against endothelial senescence, SIRT1 also plays a key role in preventing stress-induced premature senescence (SIPS) and lung inflammaging, one of the hallmarks of chronic obstructive pulmonary disease (COPD) or emphysema (Yao et al., 2012). The levels of SIRT1 expression and activity are significantly reduced in the lungs of COPD patients. In addition, enhancement of SIRT1 expression or activity inhibits stress-induced premature cellular senescence and also provides protection against cigarette smoke-induced emphysema (Yao et al., 2012). This SIRT1-mediated protection against emphysema is reported to be catalyzed by FOXO3 transcription factor (Yao et al., 2012).

## Replicative senescence

Replicative senescence is brought about in primary cells because of telomere shortening after every round of cell cycle, which signals the cells of stress and limits the number of cell divisions. Expression level of SIRT1 is reported to decrease as the primary human fibroblasts enter replicative senescence phase (Michishita et al., 2005). Additionally, SIRT1 overexpression is reported to attenuate oncogene-induced replicative senescence via p53 deacetylation (Langley et al., 2002). However, upon prolonged cell replication in culture, primary *Sirt1* ^-/-^ mouse embryonic fibroblasts (MEFs) display resistance towards replicative senescence (Chua et al., 2005). Nevertheless, SIRT1 knockdown in human diploid fibroblasts has been reported to induce cell proliferation (Abdelmohsen et al., 2007). The contribution of SIRT1 in promoting replicative senescence can be further gauged from the finding that it deacetylates histone H1 on lysine 26 (K26), and also histone H4 at K16 and H3 on K9, to endorse facultative heterochromatin formation (Vaquero et al., 2004). It is widely known that loss of heterochromatin is one of the hallmarks for premature cellular senescence and accelerated aging (Shumaker et al., 2006). Hence, given the progressive decline in SIRT1 level with passage, it is plausible that these histone marks get hyperacetylated to further lead to heterochromatin loss, thus resulting in eventual replicative senescence. Moreover, SIRT1 is reported to deacetylate WRN, the protein mutated in Werner syndrome causing telomere attrition (Li et al., 2008). Taken together, SIRT1 plays a key role in blocking replicative senescence via regulation of multiple proteins.

## Maintenance of ‘stemness’

SIRT1 expression levels have been reported to be higher in mouse embryonic stem cells (mESCs) as compared to the differentiated cells (Saunders et al., 2010). This phenomenon has been attributed to post-transcriptional downregulation of SIRT1 by a range of miRNAs, such as miR-9, miR-181a and b, and others (Saunders et al., 2010). Interestingly, the authors also reported an induction in SIRT1 protein levels when the MEFs were reprogrammed into induced pluripotent stem (iPS) cells. Recently, SIRT1 has been implicated in the maintenance of mESCs self-renewal by being directly regulated viamiR-29b in response to reactive oxygen species (ROS) (Xu et al., 2014). In addition, SIRT1 has been reported to be involved in the maintenance of haematopoietic stem cells (HSCs) homeostasis and lineage specification, where loss of SIRT1 in HSCs garnered DNA damage, accumulation of age-linked molecules and other phenotypes of aging HSCs (Rimmelé et al. 2014).Intriguingly, overexpression of SIRT1 or its activation by resveratrol is reported to rescue senescence-associated phenotypes and also angiogenic defects in preterm-endothelial colony forming progenitor cells (PT-ECFCs) (Vassallo et al., 2014). To add to this, overexpression of SIRT1 also delays senescence in the bone marrow-derived mesenchymal stem cells (B-MSCs) (Yuan et al., 2012). Further, cleavage of SIRT1 by cathepsin triggers stress-induced premature senescence (SIPS) in endothelial progenitor cells (Chen et al., 2012). On the whole, SIRT1 stands out as a prominent housekeeper of maintaining stemness by resisting senescence in the cells.

## DNA damage repair and aging

Impaired DNA damage repair and accrued genomic instability are some of the chief factors contributing to premature cellular senescence and accelerated aging (Liu et al., 2005). In this arena, SIRT1 is reported to induce DNA damage repair by deacetylating the repair protein Ku70 upon irradiation-induced DNA damage (Jeong et al., 2007). The association of RNA-binding protein HuR, SIRT1 and NBS1 in the maintenance of genomic integrity upon genotoxic stress is also extensively studied (Gorospe and de Cabo, 2008). SIRT1 has been linked in safeguarding against DNA damage and atherosclerosis in human vascular smooth muscle cells (VSMCs) by activating the repair protein Nijmegen breakage syndrome-1 (NBS-1), but not p53 (Gorenne et al., 2013). SIRT1 is also widely acknowledged to deacetylate PARP1, a key player in DNA damage repair and aging, and thus mediate response signaling upon genotoxic stress (Rajamohan et al., 2009). The intricate inter-linkage between SIRT1 and PARP1 has been time and again established to play crucial roles in genotoxic stress and DNA damage responses (Luna et al., 2013) The involvement of SIRT1 in DNA damage repair has been further solidified by the finding that it activates homologous recombination (HR) repair in human cells (Uhl et al., 2010). However, the authors refuted the involvement of Ku70, PARP1 or NBS1 in SIRT1-mediated repair, and advocated the role of WRN protein in this process. This suggests that SIRT1 possibly mediates different repair pathways via modulation of repair proteins in a mutually exclusive way. On the other hand, overexpression of SIRT1 has been reported to negatively regulate Tip60-mediated acetylation of H2AX, an event finely regulated to elicit proper DNA damage response (Yamagata and Kitabayashi, 2009). Thus, SIRT1 possibly represses extensive DNA damage response to fine-tune HR repair process by repressing H2AX over-activation. Recently, SIRT1 has been reported to display prompt recruitment to double strand breaks (DSBs) in post-mitotic neurons, where it deacetylates HDAC1 and stimulates ATM autophosphorylation to mediate non-homologous end joining (NHEJ) repair (Dobbin et al., 2013). Thus, SIRT1 plays a protective role in maintaining genomic integrity not only against genotoxic stress but also to attenuate neurodegeneration.

## Lifespan extension

*SIR2* was the first identified evolutionarily conserved gene which had been implicated in the expansion of lifespan. Attention was drawn towards the sirtuins when sir2 inactivation in yeasts reduced lifespan by 50%, while introduction of an extra copy of the gene *SIR2* increased yeast lifespan by 30% (Kaeberlein et al., 1999). This finding has been contradicted by an independent research group which failed to replicate longevity expansion in yeast or drosophila by sir2 overexpression (Burnett et al., 2011). However, another group could successfully extend longevity in *C. elegans* by *sir-2.1* overexpression, attributing this occurrence to nicotinamide (NAM) methylation (Schmeisser et al., 2013). Apart from these reports, *Sirt1* transgenic mice did not exhibit any overall lifespan extension (Herranz et al., 2010). However, SIRT1 overexpression in those mice displayed improvement of some age-associated phenotypes, such as wound healing, decreased tumorigenicity, better maintenance of glucose homeostasis and others. Intriguingly, SIRT1 overexpression specifically in brain increases longevity by approximately 11% in both male and female mice, and also reduces incidence of cancer (Satoh et al., 2013). This phenomenon has been largely attributed to SIRT1-mediated upregulation of Orexin type-2 receptor (*Ox2r*) expression specifically in the nuclei of dorsomedial and lateral hypothalamic (DMH and LH) regions of brain. In addition, this organ-specific SIRT1 activation can be explained by the observation that NK2 homeobox 1 (Nkx2-1) specifically colocalizes with SIRT1 in the DMH and LH regions of brain to upregulate Ox2r expression and thus increase longevity. Hence, this study further reinstated the importance of SIRT1 in delaying senescence and premature aging. Nevertheless, loss of sirt1 in mice resulted in severe growth defects causing death of litters in the late prenatal or early postnatal stages, thus implying SIRT1’s major contribution in development (Cheng et al., 2003). Given the involvement of SIRT1 in deacetylating and deactivating p53, the authors also observed that p53 was hyperacetylated upon DNA damage in *Sirt1* ^-/-^ mice tissues (Cheng et al., 2003). However, mice deficient for both sirt1 and p53 neither displayed any significant differences from *Sirt1* ^-/-^ mice, nor any amelioration in the growth and developmental defects (Kamel et al., 2006), thus suggesting that the impairments observed in *Sirt1* ^-/-^ mice are not majorly attributable to p53 activation. Recently, SIRT1 activation has been linked to amelioration of skeletal muscle performance in sarcopenia or atrophy during aging, mainly via inhibition of PARP1 (Mohamed et al., 2014). Taken together, SIRT1 can be regarded as a prominent, although much debated, player in promoting longevity and attenuating premature aging.

## Involvement in laminopathy-based premature aging

Animal models of laminopathy-based premature aging provide excellent model systems for studying the process of accelerated aging and cellular senescence. Although rare, some of the disease phenotypes are highly severe with irreparable mortality in patients in their early teens, such as in Hutchinson-Gilford Progeria Syndrome (HGPS) (Schreiber and Kennedy, 2013). Loss of ZMPSTE24, the metalloproteinase responsible for cleaving the precursor prelamin A into mature lamin A, reiterates progeroid phenotypes in mice (Navarro et al., 2004). Our recent work has further established the role of SIRT1 in delaying premature cellular senescence and aging by being involved in laminopathy-based premature aging (Liu et al., 2012). We reported that lamin A, a nuclear lamina protein, interacts with SIRT1 and also activates it (Liu et al., 2012). This interaction is further induced by resveratrol, thus resulting in ultimate activation of the sirtuin protein. Our study provided a mechanistic explanation to the long debated question of whether or not resveratrol aids in SIRT1 activation (Kulkarni and Cantó 2014). We also observed a prominent decline of adult stem cells (ASCs) in the tissues derived from *Zmpste24* ^-/-^ mice, which was primarily attributable to the dissociation of SIRT1 from nuclear matrix in the presence of prelamin A or progerin (the precursor and mutant forms of lamin A respectively). Interestingly, treatment with resveratrol not only rescued ASC attrition, but also extended lifespan in the mutant mice along with reduction of body weight loss and improvement in bone mineral density. This study also reinforced the role played by SIRT1 in the maintenance of stem cells. However, it remains to be seen whether SIRT1 also plays significant roles in delaying senescence or increasing lifespan in other models of laminopathy-based premature aging.

## Diverse roles in other diseases

There are multiple disorders which majorly contribute to the occurrence of premature aging, such as metabolic dysregulation, neurodegenerative diseases, inflammation and also tumorigenesis (Giblin et al., 2014). SIRT1 has been ascribed multifaceted roles in the regulation of proper metabolic homeostasis by maintaining lipogenesis, gluconeogenesis, fatty acid oxidation and others (Chang and Guarente, 2014). Caloric restriction-mediated lifespan extension is also largely attributed to SIRT1 (Chen and Guarente, 2007). So far neurodegenerative disorders are concerned, SIRT1 has been reported to deacetylate tau, the protein causing Alzheimer’s disease upon pathogenic aggregation, and trigger its degradation via ubiquitin (Min et al., 2010). Also, overexpression of SIRT1 is reported to inhibit accumulation of α-synuclein, whose pathogenic aggregation in neurons causes Parkinson’s disease, and thus leads to longevity (Donmez et al., 2012). Recently, diminished SIRT1 activity has been attributed to be one of the chief reasons for causing Cockayne syndrome, a premature aging disorder characterized by severe neurodegeneration (Scheibye-knudsen et al., 2014). SIRT1 has also been intricately linked to lung inflammaging where it is reported to play significant anti-aging roles (Yao et al., 2012). However, in regard to cancer, SIRT1 plays both tumor suppressive and oncogenic roles (Yuan et al., 2013).

The immense importance of SIRT1 in the process of anti-aging can be further ascertained from the implementation of multiple small molecule drugs which primarily act as SIRT1 activators (Hubbard and Sinclair, 2014). On the whole, SIRT1 can be regarded as a crucial anti-aging protein which mediates its widespread effects in preventing premature senescence and accelerated aging by regulating multiple molecular pathways.

## SIRT2

SIRT2 is the second mammalian sirtuin protein which is emerging out as a potential target in preventing age-related disorders. Its possible involvement in the process of aging and longevity can be fathomed from the observation that expression level of SIRT2 was found upregulated in kidney and white-adipose tissues of caloric-restricted mice (Zhu et al., 2012). However, it remains to be seen whether caloric-restriction fails to extend lifespan in mice lacking Sirt2. This will provide direct evidence of the involvement of SIRT2 in this dietary intervention-mediated lifespan extension. SIRT2 was initially identified to deacetylate α-tubulin at lysine 40 and histone H4 at lysine 16 (de Oliveira et al. 2012). Apart from these, SIRT2 is also known to deacetylate forkhead transcription factors of class O, namely FOXO1 and FOXO3 (Wang and Tong, 2009, Wang et al., 2007). Given the widely acknowledged functions of FOXO transcription factors in a spectrum of pathways directly or indirectly linked to aging, such as metabolic regulation, DNA damage repair, apoptosis and others, it is tempting to speculate that SIRT2 may play an intricate role in anti-aging processes. Moreover, an increasing proportion of evidence suggests tumor-suppressive functions of SIRT2 (de Oliveira et al. 2012). Since increasing incidence of cancer is associated with progressive aging, SIRT2 can be considered as a potential anti-aging protein. However, further experimentation is required to confirm this model. Recently, SIRT2 has been implicated in the suppression of glioma formation (Li et al., 2013). In this study, the authors identified p65 (a subunit of NF-ĸB signaling) as a target of deacetylation by SIRT2 at lysine 310, thereby inhibiting miR-21 transcription via blockage of p65 binding with its promoter region. This report further supported the role of SIRT2 in tumor suppression and opened up new avenues for the involvement of SIRT2 in premature aging via regulation of NF-ĸB signaling. Additionally, SIRT2 overexpression in BubR1 mice, which harbour hypomorphism for BubR1 and display premature aging phenotypes, has been reported to extend lifespan in the mutant mice (North et al., 2014). Given the many implications of Wnt signaling in tumorigenesis and aging, SIRT2-mediated attenuation of Wnt signaling pathway upon oxidative stress (Nguyen et al., 2014), further insinuates at the involvement of this sirtuin in the regulation of aging via multiple mechanisms. Apart from these, SIRT2 is also ascribed significant roles in safeguarding against neurodegenerative disorders, such as Parkinson’s and Huntington’s diseases (de Oliveira et al., 2012). Thus, SIRT2 can be considered as a promising target in designing intervention against premature aging.

## SIRT3

SIRT3, predominantly located in mitochondria, has been primarily linked to the regulation of a variety of mitochondrial processes, such as β-oxidation, ATP generation, management of reactive oxygen species (ROS) and others (Giblin et al., 2014). However, its roles in aging-associated disorders have begun emerging in the recent years. Although *Sirt3*-knockout mice do not exhibit any severe signs of premature aging or tumorigenesis, the mutant mice do display glucose intolerance, dysregulated ROS production, and heightened white adipose tissue (WAT) development (Jing et al., 2011). Since ROS-induced oxidative stress is one of the critical contributors of premature cellular senescence, it is possible that SIRT3 might have a hand in regulating this process. SIRT3 can be strongly associated with the maintenance of metabolic regulation based on the finding that loss of *Sirt3* in the germline of mice leads to accelerated development of obesity, hepatic steatosis and insulin resistance upon high-fat diet (HFD) feeding (Hirschey et al., 2011). This study also identified reduction in Sirt3 expression level when mice were fed with high-fat diet. Recently, SIRT3 has been implicated in the maintenance of regenerative capacity in the haematopoietic stem cells with progressive age (Brown et al., 2013). Haematopoietic stem cells display high expression levels of SIRT3, where this sirtuin regulates mitochondrial protein acetylation profiles and blocks oxidative stress generation. This groundbreaking discovery not only recognized the significant roles of SIRT3 in maintaining stemness, but also laid out a path for further dissecting its roles in stem cell-based intervention for metabolic disorders resulting in premature aging. In addition, some single nucleotide polymorphisms (SNPs) in *Sirt3* gene have been attributed in the extension of longevity (Giblin et al., 2014). Also, several lines of evidence suggest modulating roles for SIRT3 in response to caloric-restriction (Kincaid and Bossy-Wetzel 2013). Apart from these, a recent study elucidated the involvement of SIRT3 in protection against neurodegeneration in Huntington’s disease (Fu et al., 2012). The study identified reduced expression of SIRT3 because of mutant protein huntingtin (Htt), which causes Huntington’s disease. Treatment with viniferin, a naturally occurring compound, ameliorated the disease phenotypes by enhancing SIRT3 expression level (Fu et al., 2012). Taken together, these studies clearly reinstate the importance of SIRT3 in the maintenance of metabolic regulation, regeneration in stem cells, and also neuroprotection, all of which contribute directly or indirectly in the acceleration of aging in individuals when dysregulated.

## SIRT4

SIRT4 is the second mitochondrial sirtuin protein, with no reported deacetylase activity (Saunders and Verdin 2007). However, this sirtuin possesses ADP-ribosyltransferase activity towards glutamate dehydrogenase (GDH) (Haigis et al., 2006). This study also identified reduction in SIRT4 level upon caloric-restriction, thus suggesting that SIRT4 might antagonize this dietary restriction-mediated effect, unlike SIRT1 and SIRT3. Further, SIRT4 has been implicated in regulating lipid metabolism by attenuating peroxisome proliferator-activated receptor α (PPARα) and thus repress rates of fatty acid oxidation in the hepatocytes (Laurent et al., 2013). SIRT4 has been recently identified to maintain mitochondrial ATP homeostasis (Ho et al., 2013). Although, there has been no direct evidence relating SIRT4 activities with the aging process, the involvement of this sirtuin in mitochondrial biogenesis and ATP production insinuate at a faint possibility that SIRT4 might be linked to the process of aging.

## SIRT5

SIRT5 is the third mitochondrial sirtuin protein with very few substrates identified till date. It was first identified to deacetylate carbamoyl phosphate synthetase 1 (CPS1) and thus regulate the urea cycle (Nakagawa and Guarente 2009). It has been assumed that SIRT5 might also play a role in mitochondrial metabolism in response to varying nutritional states (Shih and Donmez, 2013). Lately, SIRT5 has been confided significant desuccinylase activity in mitochondria (Rardin et al., 2013), implicating the role for SIRT5 in the repression of ketogenesis by desuccinylation of the rate-limiting enzyme 3-hydroxy-3-methylglutaryl-CoA synthase 2 (HMGCS2). Since mitochondrial dysregulation plays a key role in bringing about senescence phenotypes, it is not impossible that SIRT5 might play a regulatory role in the process. Furthermore, polymorphisms in the promoter region of *SIRT5* gene have been associated with the development of premature aging in amygdala region of brain (Glorioso et al., 2011), suggesting the possible involvement of SIRT5 promoter polymorphisms in the incidence of Parkinson’s disease. Further studies are required in order to clearly differentiate roles (if any) of this sirtuin in the process of accelerated aging.

## SIRT6

SIRT6 is predominantly a nuclear protein, whose association with chromatin has time and again been established (Kugel and Mostoslavsky 2012). SIRT6 was primarily identified as a histone deacetylase with specificity towards histone H3 at lysine 9 and 56 (K9 and K56) (Michishita et al., 2008, Michishita et al., 2009). This sirtuin gained prominence when its knockout mouse model developed severe premature aging phenotypes with mortality resulting within a month (Mostoslavsky et al., 2006). Moreover, SIRT6 is the only mammalian sirtuin which displayed clear increase in lifespan when overexpressed in the whole body of mice (Kanfi et al., 2012). Intriguingly, this longevity extension has only been observed in male mice, and the underlying mechanism of this gender-specificity is still under debate. SIRT6 can be regarded as an important anti-aging protein with multifaceted roles in DNA damage repair, metabolic regulation, inflammation and also tumor suppression, as discussed below in details.

## Maintenance of genomic stability

The premature aging phenotypes observed in the *Sirt6* ^-/-^ mice were primarily attributed to the defects observed in base excision repair (BER) machinery (Mostoslavsky et al., 2006). This seminal finding ignited interest in the scientific community and multiple roles of SIRT6 in DNA damage response began to be deciphered gradually. For example, SIRT6 is identified to deacetylate CtIP, a DNA end resection protein, and mediate homologous recombination (HR) repair (Kaidi et al., 2010). SIRT6 also mono ADP-ribosylates PARP1 in response to oxidative stress and thus promotes non-homologous end-joining (NHEJ) repair process (Mao et al., 2011). In addition, SIRT6 is one of the prompt recruits to DNA double strand break (DSB) sites, and further helps in the localization of other repair proteins like DNA-PKcs, SNF2H and others (McCord et al., 2009, Toiber et al., 2013). SIRT6 was also observed to progressively decline with passage in human fibroblasts (Mao et al., 2012). In addition, overexpression of this sirtuin alleviated the repression of HR repair observed during replicative senescence. SIRT6 has been also reported to localize in telomeric chromatin where it deacetylates histone H3 at K9, thus stabilizing the interaction of WRN protein and maintaining proper telomeric metabolism (Michishita et al., 2008). Since impairment in DNA damage repair and telomere shortening are some of the hallmarks of premature cellular senescence and are critical contributing factors for accelerated aging, SIRT6 can be considered as a pivotal player in DNA damage repair-mediated anti-aging process.

## Metabolic homeostasis

SIRT6 is a major regulator of the maintenance of glucose homeostasis. It has been reported to repress gluconeogenesis in hepatocytes by regulating GCN5-PGC1α pathway, thus preventing diabetic hyperglycemia (Dominy et al., 2012). SIRT6 acts as a co-repressor of the transcription factor Hif1α (which is involved in sensing nutritional state of cells) and also deacetylates H3K9 at the promoters of several glycolytic genes (Zhong et al., 2010). This provides a mechanistic explanation to the lethal hypoglycemia observed in *Sirt6* ^-/-^ mice. SIRT6 has been also related in regulation of lipogenesis by repression of lipogenic transcription factors, SREBP1 and SREBP2 via multiple mechanisms (Elhanati et al., 2013). This study identified reduced levels of low-density lipoprotein cholesterol in the *Sirt6* transgenic mice, thus providing another mechanistic explanation to the increased longevity in the mutant mice. The involvement of SIRT6 in fat metabolism is further bolstered by the finding that loss of this sirtuin in the liver of mice results in formation of fatty liver due to upregulated glycolysis and triglyceride synthesis (Kim et al., 2010). SIRT6 is also linked in attenuating insulin-like growth factor (IGF)-AKT signaling and thus preventing cardiac hypertrophy (Sundaresan et al., 2012). On the whole, SIRT6 is a critical housekeeper of metabolic homeostasis, the imbalance of which eventually results in premature aging phenotypes.

## Tumor suppression

SIRT6 has been identified as a vital tumor suppressor which critically regulates cancer metabolism (Sebastián et al. 2012). This study also identified downregulation of SIRT6 in several forms of human cancer, such as pancreatic and colorectal cancer. Moreover, overexpression of SIRT6 in non-small cell lung cancer (NSCLC) cells radiosensitizes them which inhibits their proliferation, further supporting the idea of SIRT6 being a tumor suppressor (Cai et al., 2014). However, this concept has been contradicted by an independent research group which reported that depletion of SIRT6 in mouse skin inhibits tumorigenesis (Ming et al., 2014). This is suggestive of particular cancer cell-type specific activities of SIRT6. Nevertheless, the finding that overexpression of SIRT6 triggers heightened apoptotic response specifically in cancerous cells but not in normal cells (Van Meter et al., 2011), further reinstates the role of this sirtuin as a potent tumor suppressor. Also, degradation of SIRT6 by MDM2 has been observed in multiple lines of breast cancer cells (Thirumurthi et al., 2014). All these findings solidify the concept of SIRT6 being a key tumor suppressor, and further strengthen its role in delaying aging, since cancer incidence and progressive aging go hand in hand.

## Inflammation

In some recent reports, SIRT6 has been identified to possess anti-inflammatory functions. For example, loss of SIRT6 in human umbilical vein endothelial cells (HUVECs) induced proinflammatory cytokine expression, such as interleukin 6 (IL-6), IL-8 and also IL-1β (Lappas, 2012). Moreover, overexpression of SIRT6 in human rheumatoid arthritis fibroblast-like cells and also in mice with collagen-induced arthritis attenuated proinflammatory cytokine levels (Lee et al., 2013). Given the huge implications of NF-ĸB in inflammatory responses, the finding that SIRT6 attenuates NF-ĸB signaling by deacetylating histone H3 at K9 on the promoters of NF-ĸB target genes (Kawahara et al., 2009), further establishes the role of SIRT6 as a critical anti-inflammatory protein. Also, loss of Sirt6 in immune cells derived from mice display chronic liver inflammation and fibrosis (Xiao et al., 2012). However, another study has advocated that SIRT6 induces the secretion of cytokines and might possibly be involved in pro-inflammatory responses (Bauer et al., 2012). Since inflammatory responses have been linked to the development of premature aging phenotypes, the role of SIRT6 in repressing age-associated pathologies get strengthened more and more.

Taken together, SIRT6 stands out as a key modulator of anti-aging processes which mediates its regulatory roles via multiple pathways to delay cellular senescence and accelerated aging. Hence, identification of SIRT6 activators can have huge therapeutic impact in designing intervention for premature aging disorders and other age-associated pathologies.

## SIRT7

SIRT7 is the seventh mammalian sirtuin protein predominantly localized in the nucleolus with an established role in rDNA transcription activation (Kim and Kim, 2013). Increasing evidences are surfacing regarding the functioning of this sirtuin which are gradually hinting its potential role in premature aging. Inactivation of the *Sirt7* gene in mice results in lifespan reduction and the mutant mice also develop cardiac hypertrophy and inflammatory cardiomyopathy (Vakhrusheva et al. 2008a, b). These phenotypic outcomes have been primarily attributed to lack of Sirt7-mediated p53 deacetylation in the mutant mice, thus resulting in p53 hyperactivation and apoptosis. However, no direct evidence has been provided by the authors at this end regarding *in vivo* Western blotting or exact sites of p53 deacetylation by SIRT7. Thus, further experiments need to be performed in order to draw a clear picture. Nevertheless, SIRT7 has been ascribed an anti-proliferative role since its overexpression ceases tumorigenicity in several lines of murine cells (Vakhrusheva et al. 2008a). In contrast to this observation, SIRT7 has been characterized as an oncogene which selectively deacetylates histone H3 at lysine 18 to maintain tumorigenic potential in cancer cells (Paredes et al., 2014). Also, SIRT7 expression level is found heightened in a large number of patients with human hepatocellular carcinoma (HCC), and knockdown of SIRT7 repressed growth in liver cancer cells (Kim et al., 2013). Apart from the recognition of being an oncogene, SIRT7 is also implicated in the aging process since its expression levels were observed to be reduced in senescent cells (Lee et al., 2014). Moreover, the involvement of SIRT7 in attenuation of ER stress and prevention of fatty liver disease formation (Shin et al., 2013), further extend its roles in maintenance of metabolic regulation. SIRT7 is also observed to regulate lipid metabolism in the liver (Yoshizawa et al., 2014). On the whole, it would not be inappropriate to speculate potential roles of SIRT7 in maintaining metabolic homeostasis, cancer metabolism, and thus anti-aging process.

## Conclusion

Till date, substantial number of evidences has laid foundation to the concept of sirtuins being potent anti-aging proteins. Their multivalent roles in delaying cellular senescence and blocking the development of premature aging phenotypes further mark them as promising targets to design intervention for several age-associated pathologies (Table 1). SIRT1, being the most extensively experimented sirtuin with regards to aging and longevity, has triggered interest in scientific communities to develop small molecule activators or drugs in amelioration of a wide range of aging disorders. Several such activators and drugs for SIRT1 activation are also commercially available for testing in labs (Mellini et al., 2014). For example, the anti-aging effects of resveratrol are primarily attributed to SIRT1 activation. With the emerging reports on potential anti-aging effects of the other sirtuins, specifically SIRT6, it would be important to screen out the activators or modulators of these sirtuins and design drugs to test their effects in the animal models of premature aging. Identification of such potent drugs to stimulate sirtuin functioning can prove to be breakthroughs in ameliorating age-associated disorders. Although development of such small molecule activators of sirtuins or drugs and their inclusion in clinical trials is a long-shot process, the increasing number of findings elaborating the various sirtuins-mediated anti-aging mechanisms, abridge the path.In conclusion, the SIRT proteins can certainly be regarded as essential factors in delaying cellular senescence, repressing premature aging and enhancing longevity and healthspan in the living systems.

**Table 1:** Involvement of sirtuins in the pathways contributing to premature aging when dysregulated

Pathways contributing to premature aging	SIRT1	SIRT2	SIRT3	SIRT4	SIRT5	SIRT6	SIRT7
Cellular/replicative senescence	√	?	**	?	?	√	?
Maintenance of stem cells	√	**	√	?	?	√	?
Maintenance of genomic integrity	√	**	?	?	?	√	**
Metabolic regulation	√	√	√	√	√	√	√
Regulation of tumorigenesis	√	√	√	?	?	√	√
Inflammation	√	√	**	**	?	√	√
Neurodegeneration	√	√	√	√	**	?	?

√: Established roles, **: possible roles, **?:** No reported roles

## Abbreviations

ATM: ataxia telangiectasia mutated
ATP: adenosine triphosphate
CtIP: C-terminal binding protein interacting protein
ER: endoplasmic reticulum
HCC: hepatocellular carcinoma
HDACs: histone deacetylases
HSC: haematopoietic stem cell
IGF: insulin-like growth factor
MEF: mouse embryonic fibroblast
NAD: nicotinamide adenine dinucleotide
PARP1: poly [ADP ribose] polymerase 1
SIPS: stress-induced premature senescence
SIRT: silent information of regulator
ZMPSTE24: zinc metalloproteinase Ste24
